# Visual and vestibular motion perception in persistent postural-perceptual dizziness (PPPD)

**DOI:** 10.1007/s00415-024-12255-x

**Published:** 2024-03-05

**Authors:** Renana Storm, Janina Krause, Smila-Karlotta Blüm, Viktoria Wrobel, Antonia Frings, Christoph Helmchen, Andreas Sprenger

**Affiliations:** 1 Department of Neurology, University Hospital Schleswig-Holstein, University of Lübeck, Campus Lübeck, Ratzeburger Allee 160, 23538 Lübeck, Germany; 2 Center of Brain, Behavior and Metabolism (CBBM), University of Lübeck, Ratzeburger Allee 160, 23562 Lübeck, Germany; 3 Institute of Psychology II, University of Lübeck, Ratzeburger Allee 160, 23562 Lübeck, Germany

**Keywords:** Persistent postural-perceptual dizziness, PPPD, Motion perception, Vestibular, Functional disorder

## Abstract

Persistent postural-perceptual dizziness (PPPD) is a chronic disorder of perceived unsteadiness. Symptoms can be exacerbated in visually complex stationary or moving environment. Visual dependence and increased motion sensitivity are predictors for PPPD but its pathophysiology remains unknown. We hypothesized an abnormal sensory–perceptual scaling mechanism in PPPD and tested visual- and vestibular perceptional thresholds in 32 patients and 28 age-matched healthy control subjects (HC). All participants showed normal vestibular function tests on quantitative testing. Visual motion coherence thresholds were assessed by random dot kinetomatograms. Vestibular perceptional thresholds of egomotion were assessed by binaural galvanic vestibular stimulation (GVS) and passive chair rotation around an earth-vertical axis. Chair rotation trials were contrasted with no-motion (sham) stimulus trials. Mean thresholds of visual motion perception were higher in patients compared to HC*.* The perception threshold of GVS was lower in patients but the threshold of correctly perceived egomotion during chair rotation did not differ. Interestingly, the number of trials with correct perception in the no-motion condition increased with the threshold of correct responses for rotatory egomotion in patients. Unlike expected, PPPD patients required more coherently moving random dots than HC to perceive visual motion. A poorer complex visual motion recognition, e.g., traffic visual stimuli, may increase anxiety and levels of uncertainty as visuomotor reactions might occur delayed. The vestibular rotatory perception threshold predicted the probability of making false assignments in the sham condition in PPPD, i.e., patients who readily recognize the correct egomotion direction are prone to perceive egomotion in the no-motion condition. As this relation was not found in healthy subjects, it may reflect an abnormal sensory–perceptual scaling feature of PPPD.

## Introduction

Persistent postural-perceptual dizziness (PPPD) is a chronic (> 3 months) disorder of perceived unsteadiness in the absence of peripheral sensory abnormalities or structural brain abnormalities [1]. The Bárány Society has established diagnostic criteria in 2017 [2]. Symptoms are often exacerbated by active or passive motion or exposure to moving visual stimuli.

While primary PPPD lacks precipitating factors [3], secondary PPPD is often triggered by a previous event destabilizing posture, e.g., episodic (vestibular neuritis) or recurrent vestibular disorders (benign positional paroxysmal vertigo, BPPV), syncope, or prolonged physiological multisensory stimulation (Mal de debarkment syndrome) [4]. Importantly, perceptual unsteadiness and dizziness persists despite complete recovery of abnormal sensory (vestibular) function or the deliberation from abnormal vestibular excitation (BPPV). It is not related to the magnitude of previous or still persistent vestibular dysfunction [5]. This suggests that PPPD results from a maladaptation of perceived postural control in response to a misprediction of the sensory consequence of one’s own movements or even anticipated actions. This misjudgement may result from altered thresholds of sensory (visual, vestibular, proprioceptive) motion perception. Among various sensory signals, PPPD patients are thought to rely more on visual inputs [6, 7] compared to other sensory, i.e., vestibular and somatosensory, inputs to stabilize posture and balance. Sensitivity to moving visual stimuli becomes annoying in PPPD, a symptom that subjects did not experience before the precipitating event.

Several data indicate altered visual sensitivity, examined, e.g., by the Visual Vertigo Analog Scale, imaging brain activity in response to virtual reality visual moving scenes or the Rod-and-Disc test [8, 9]. However, neither visual nor vestibular motion sensitivity has been systematically examined in PPPD on a perceptual level. We have previously shown that thresholds of motion perception in response to biaural galvanic vestibular stimulation are lower in PPPD compared to healthy control subjects [10]. Vestibular perception thresholds in response to passive vestibular turntable motion seem to be lower than in healthy subjects but this study was neither controlled for the perceived motion direction nor by sham stimuli [11]. In this study, we investigated visual motion perception in PPPD patients and healthy controls via a random-dot-kinematogram and vestibular motion perception via various vestibular turntable chair conditions. We used perceptional thresholds during binaural galvanic vestibular stimulation (GVS) as an active comparator and during no-motion (sham) trials as a control condition. We expected lower visual and vestibular motion perception thresholds in PPPD patients.

## Methods

### Participants

Thirty-two PPPD patients and 28 age-matched healthy control subjects (HC) were included in this study. All participants were right-handed (Edinburgh Handedness Inventory [12]. Based on a related study revealing significant group differences [11] we calculated a cohort size by using a power analysis revealing 18 participants per group (G*Power 3.1.9.7 [13]; effect size 0.96, alpha probability of 0.05, power of 0.8). All patients met the diagnostic PPPD criteria of the Barany Society [2]. Healthy subjects had no history of vertigo, dizziness, migraine, or other types of balance disorders. Demographics and patient characteristics are summarized in Table 1, including questionnaires addressing motion sickness susceptibility, dizziness intensity, impact on daily life, level of anxiety, depression, and personality features. We used the validated Niigata Questionnaire on PPPD [14], the Motion Sickness Susceptibility Questionnaire (MSSQ) [15]; the Neuroticism and Extraversion scores of the 5-Factor Inventory Personality Questionnaire (NEO-FFI) [16], anxiety and depression scores of the Hospital Anxiety and Depression scale (HADS) [17, 18], a questionnaire distinguishing PPPD symptoms in the context of egomotion, rest, head motion, and visual motion, i.e., the Athens-Lübeck-Questionnaire on PPPD (ALQ) [19], and the State and Visual Analog Values of the EQ-5D-3L [20]. In short, PPPD participants revealed higher values of symptom exacerbation by visual and egomotion (Niigata questionnaire for PPPD, MSSQ, ALQ), for neuroticism (NEO-FFI), and of anxiety and depression (HADS); and self-assessment for daily life quality (EQ-5D-3L). Importantly, participants were age matched as vestibular perceptual thresholds are age dependent [21]. None of the PPPD patients were on medication affecting CNS at the time of recording, specifically participants did not take any medication affecting cognition, pain or mood.

**Table 1:** Demographics, scores and patient characteristics

	PPPD (mean ± STD)	Healthy subjects (mean ± STD)	Statistical difference *p*
Number	32	28	n.s
Age (years)	43.7 ± 11.4	43.4 ± 12.6	n.s. (*p* > 0.9)
Gender (f:m)	21:11	16:12	n.s
disease duration (months)	34 ± 26	–	–
Niigata score	27 ± 13	2 ± 3	0.001
MSSQ	8 ± 7	4 ± 5	0.005
Neuroticism (NEO-FFI)	23.1 ± 8	15.1 ± 7.6	0.001
Extraversion (NEO-FFI)	26.7 ± 6.5	29.9 ± 7.1	0.075
ALQ	16 ± 7	1 ± 2	0.001
HADS-A	8 ± 4	4 ± 3	0.001
HADS-D	6 ± 4	2 ± 2	0.002
EQ VAS	64 ± 18	89 ± 11	0.001

*MSSQ* Motion Sickness Susceptibility Questionnaire, *NEO-FFI* Neuroticism and Extraversion scores of the 5-Factor Inventory Personality Questionnaire, *HADS* Hospital Anxiety and Depression scale, *ALQ* Athens-Lübeck-Questionnaire, *EQ VAS* visual analogue scale of the EQ-5D-3L

PPPD patients were recruited from the University Centre for Vertigo and Balance Disorders. Only patients with a disease duration of > 3 months were included. All participants underwent another clinical neurological and neuro-otological examination at the day of behavioral and imaging recordings. None of the participants had clinical signs of a persistent vestibular hypofunction, positional nystagmus, cerebellar dysfunction, and all of them had normal visual acuity. Exclusion criteria included persistent vestibular failure (gain < 0.7 of horizontal VOR gain, assessed by video head impulse test), dementia, major depression, personality disorders, polyneuropathy, sedative drugs, consumption of alcohol, and the inability to stand without assistance. None of the patients had abnormal vestibular functions on clinical and quantitative recordings (quantitative head impulse test, caloric irrigation, vestibular evoked myogenic potentials, subjective visual vertical) at the time of enrollment. Previous vestibular episodes included benign paroxysmal vertigo, unilateral vestibulopathy, exposure to moving platforms but clinical and vestibular function tests demonstrated complete restitution (VOR gain > 0.7) before recruitment in this study.

The study protocol was conducted in accordance with the Declaration of Helsinki and its later amendments and approved by the local Ethics Committee of the University of Lübeck (AZ 17-036, AZ 21-098) and written informed consent was obtained from all participants.

### Electrophysiological and psychophysical recordings of vestibular function

All standardized vestibular tests showed data within normal limits and no group differences. Vestibular function of participants was examined by video-oculography with caloric irrigation [bithermal cold (27°) and warm (44°) caloric irrigation] and quantitative head impulse testing (qHIT). Eye and head movements were recorded by the EyeSeeCam^®^ HIT System (Autronics, Hamburg, Germany) at a sampling rate of 220 Hz. Quantitative HIT was delivered by passive head impulses (HIT) with rapid small amplitude (10–15°) horizontal head rotations (3000–4500°/s^2^), while the participant was sitting on a chair fixating a red LED at a distance of 100 cm. For further details, see [10, 22–26]. Psychophysical perception of the visual vertical was assessed with the head fixed on a chin rest by the subject’s adjustment of a bar to the perceived visual vertical without any spatial orientation clues in a dotted half-spherical dome, which is stationary or dynamic (moving visual background) around the line of sight [27]. The normal range of SVV was defined as deviation of < 2.5°.

### Visual motion perception

Motion coherence thresholds were assessed by presenting random dot kinetomatograms (RDKs) as described by Pilz and coworkers [28, 29]. Participants were sitting 60 cm in front of a monitor (22″, Hanns.G, 60 Hz, resolution 1680 × 1050 Pixel) on which kinetomatograms were presented in a dimly lighted room via Psychophysics Toolbox [30] using Matlab^®^ (R2019b, The Mathworks Inc, Natick, MA). Participants were instructed to indicate the direction of motion after each trial as quickly and accurately as possible using keys on a standard keyboard. For each participant, we recorded percent accuracy for each condition. The RDKs consisted of 150 Gy dots, randomly assigned in size (3–9 pixels), that were presented for 400 ms in a circular black aperture of 20 cm diameter. The gray color increased in intensity with larger sizes of the dots for 3D vision. Once dots moved outside the aperture a new one was introduced with a randomly chosen direction. A distinct number of dots moved coherently in one direction (1.5°/s, 200 ms). Testing started with 80% motion coherence (the percentage of dots moving in the same direction). Its order was presented in a randomized fashion but each direction was maximally shown twice in sequence. The motion direction of each noise dot (distractor) was randomly chosen between 0° and 360°. Thresholds were reduced (by 10% above 50%, by 5% below 50% motion coherence) once the participant indicated the correct motion direction of two consecutive trials or increased in the case of two consecutive wrong directions. In the case of six consecutive wrong direction assignments, the threshold was determined by averaging the last six correct direction. Subjects received no feedback about the success of their performance. Runs were excluded if subjects failed to recognize a visual motion coherence level below 50%. After all, twenty-seven PPPD and 22 healthy participants entered the final analysis.

### Vestibular motion perception

Vestibular perception was assessed by (i) galvanic vestibular stimulation (GVS) and (ii) passive chair rotation.

**Fig. 1:**
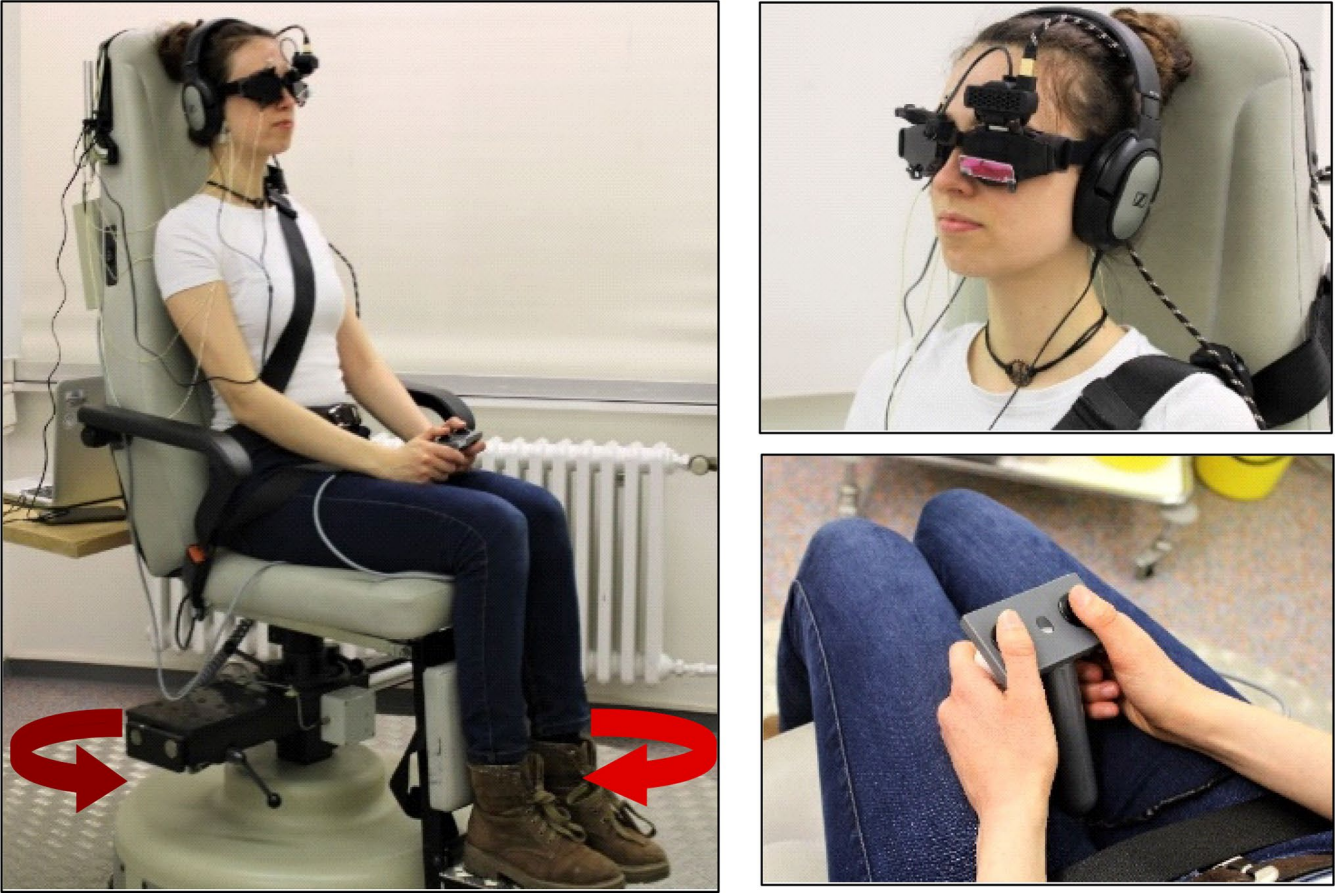
Vestibular perception threshold of chair rotation. The subject was sitting on the vestibular chair, refrained from visual and acoustic signals (see methods). The vestibular stimulus (chair rotation around the earth-vertical axis) was moving with a constant acceleration in an unpredictable direction, being randomized before (acceleration of 0.3°/s^2^). The subjects were asked to indicate the onset of passive egomotion perception by a response button once they were certain enough to determine the direction (left/right) of motion. They did not receive a feedback on whether they have indicated the right direction. They were also instructed that several trials did not deliver passive chair rotation (sham stimulation) in an irregular sequence (pseudorandomized) but that they were also asked to indicate the direction of perceived motion in case of perceived egomotion. The sham trial was stopped after 15 s

(i) We used GVS as described previously [10, 31]*.* To minimize potential nociceptive stimulation of higher GVS the stimulation site was pre-treated with local anesthetics prior the experiment (Anesderm^®^ lotion). The current stimulator (DS5 model, Digitimer Ltd., U.K.) delivered bilateral mastoid galvanic stimulation with skin contact electrodes provided by EasyCap GmbH (Herrsching/Germany). The skin surface was cleaned again and dried before contact electrodes with commercial contact paste were attached above the mastoid bilaterally. Individual sensory (vestibular) thresholds were obtained by applying 10 s 1 Hz alternating stimulation, i.e., low frequency alternating current which passed between the two mastoid electrodes. The ramp stimulus profile prevented sharp transients at stimulus onset and offset (ramp onset and offset of 100 ms duration) with a stimulation plateau of 800 ms leading to perceived head and body tilt. Threshold testing of perceived body motion started with an above threshold current (usually 1 mA) that elicited a perceived lateral to-and-fro body motion. Subsequently, starting from a low subliminal threshold (0.10 mA), the current was gradually increased in steps of 0.05 mA until the subject reported vestibular sensations, i.e., a perception of own body motion. The threshold was verified by varying the stimulation intensity until a stable threshold was found. All subjects indicated a perceived medio-lateral motion direction.
(ii) All participants were investigated while seating upright in a motorized rotary chair (Nystagliner version 3.0, Erich Jaeger GmbH + Co. KG, Friedberg, Germany), which was rotated around an earth-vertical axis with an acceleration 0.3°/s^2^ (Fig. 1) [32]. Participants were sitting in complete darkness and restrained from any visual signals by googles with obscured glasses and from room noise by providing white noise over earphones (Sennheiser HD 206, Sennheiser GmbH & Co. KG, Wedemark-Wennebostel, Germany). Participants were instructed to keep their eyes open as eye movements were recorded with video-oculography (EyeSeeCam vHIT system, recording rate: 220 Hz, Interacoustics, Germany) and additional DC-coupled horizontal electrooculography acquired at a sampling rate of 250 Hz for monitoring vigilance and vestibulo-ocular responses. Participants were instructed to indicate the direction (left vs. right) of perceived egomotion by pressing the appropriate button (left/right) on a response unit they were holding with both hands [33]. They were asked to press the button only when they are sure about the direction of perceived motion. Corrections were not possible and they did not get a feedback of correct or false responses. In case of pressing a button, rotation was stopped. Beforehand participants were also instructed about sham trials that in some (unpredictable) stimulation trials, the chair will not move at all and they should wait (no button press) until the next trial will be announced. After each stimulation trial they were asked to close their eyes during which the chair was slowly repositioned in the zero (starting) position. The verbal instruction “open your eyes” indicated that the next trial will start within a few seconds (variable jitter of 1–3 s). To get used to the stimulation and response trials, the participants were accustomed to the chair rotation in advance and had to indicate the perceived motion direction (button) and received a feedback whether the direction of motion was correct. Correct responses were required for both directions before the recording trials started. This was usually achieved within 2–4 runs. Participants had to respond to a total of 15 trials (5 trials with chair rotation to the left, 5 trials to the right, and 5 trials sham condition with no chair movement). The order of the stimuli was pseudorandomized in 4 different sets to minimize sequence effects and prediction. Each participant was randomly assigned to one of the four sets [32]. The chair acceleration was stopped by the participants by button press or when the final chair velocity of 60°/s was reached. Vestibular motion perception threshold was taken as the mean of the chair velocity (egomotion) at the time of button press.

### Statistical analysis

Statistical analyses were performed with SPSS (22.0.0.2; IBM Corp., Somer NY). THRESHOLDS of motion perception were taken as within-subject factors (repetitive runs) and GROUP (patient vs healthy controls) as between-subjects factor. Statistical comparisons were presented parametric unless stated otherwise. As some data were not normally distributed, we performed non-parametric comparisons for pairwise comparisons. For two factorial analyses we performed ANOVAs using rank transformed data. There were no significant differences between parametric and non-parametric analyses, emphasizing the robustness of ANOVAs against violations of the normal distribution assumption [34]. Multi-factorial ANOVA were performed. Significance levels of post hoc tests were Bonferroni corrected for multiple testing. Statistical differences were regarded as significant for values *p* < 0.05. Results are presented in box plots (with median, upper, and lower quartiles, e.g., 75 and 25% percentiles) for the healthy and PPPD participants. Correlation analyses were performed using Spearman-Rho coefficient unless otherwise stated.

## Results

Diagnostic workup of vestibular function (quantitative head impulse test by video-oculography—video head impulse test—of horizontal angular vestibulo-ocular reflex, caloric irrigation, subjective visual vertical using stationary and dynamic conditions, ocular vestibular evoked myogenic potentials) revealed normal vestibular function at the time of participants’ inclusion into the study.

### Visual motion perception

**Fig. 2:**
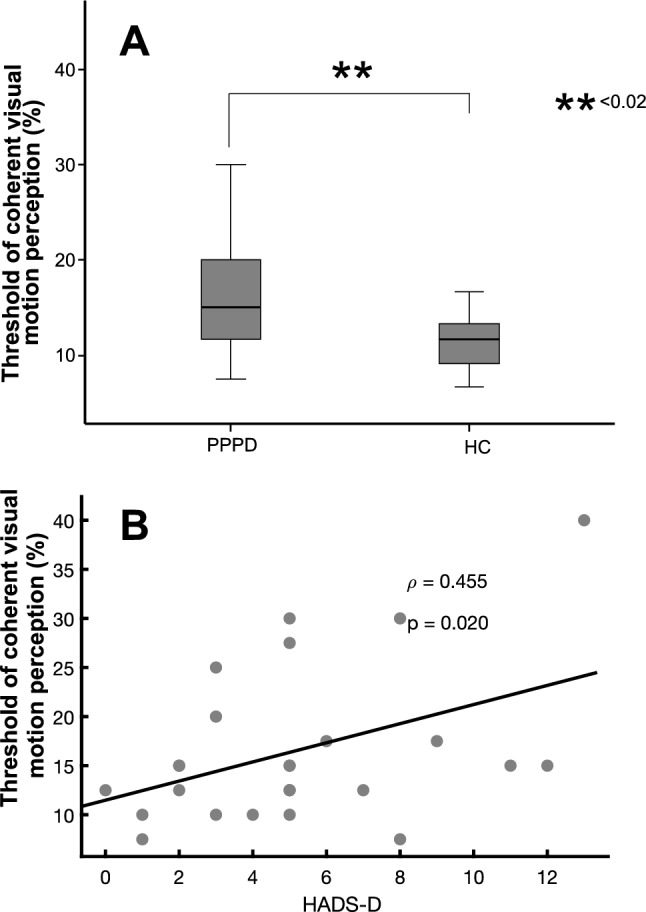
**A** Threshold of coherent visual motion perception (in %) are displayed: the threshold is significantly higher (*p* = 0.02) in PPPD compared to healthy control subjects (HC). **B** This threshold of coherent visual motion perception increased with the level of depression

Mean threshold of visual motion perception was higher in PPPD patients (mean 17.53% ± 8.7; *Z* = − 2.172, *p* = 0.030) compared to healthy control subjects (mean 11.81% ± 5.04), i.e., patients required more coherent motion of moving dots to determine the direction of coherent motion (Fig. 2A) [parametric: *F*(1,47) = 6.044, *p* = 0.018; rank transformed data: *F*(1,47) = 5.786; *p* = 0.028]. There were no within-subject differences between the consecutive 3 trials. Visual motion perception threshold did not correlate with disease duration.There was no difference between vertical or horizontal directions of coherent motion stimuli. Visual motion detection threshold increased significantly with depression, i.e., the HADS-D score (*r* = 0.432; *p* = 0.03, Fig. 2B) but did not correlate with disease duration, age, HADS-A, the severity of PPPD (Niigata PPPD score); EQ, ALQ, the Motion Sickness Susceptibility Questionnaire (MSSQ-Short), the NEO-FFI and vestibular motion perception threshold by GVS.

### Vestibular motion perception

#### Galvanic vestibular stimulation

**Fig. 3:**
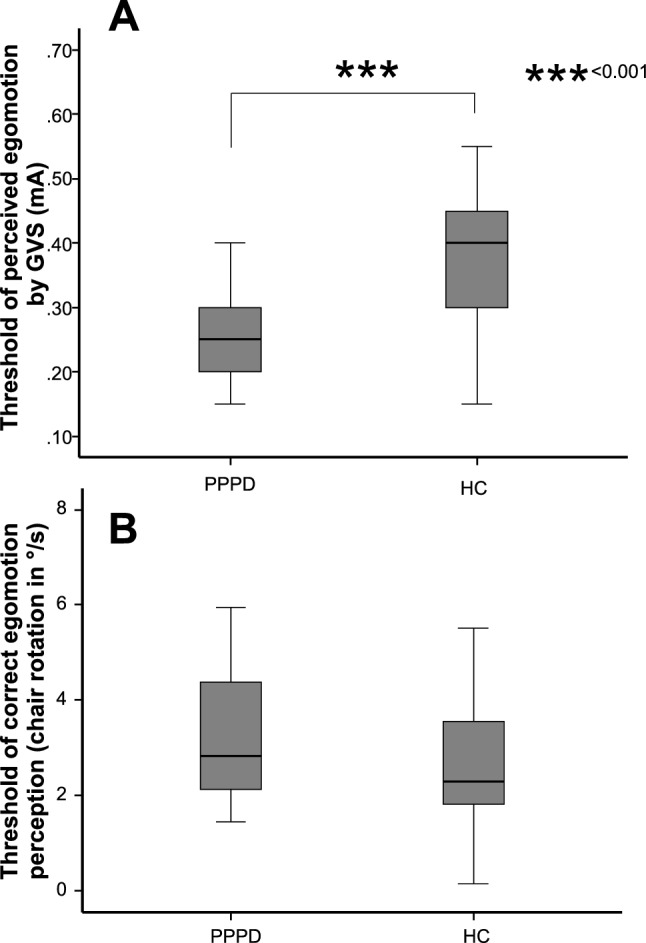
**A** Vestibular motion threshold of perceived to-and-fro sway by binaural galvanic vestibular stimulation (GVS) show significantly lower in PPPD than HC participants. **B** Threshold of correct rotatory egomotion perception during chair rotation display no group effect

The perception threshold of GVS was significantly lower in PPPD patients (0.28 ± 0.11) compared to healthy controls (0.39 ± 0.12) (*Z* = − 3.436; *p* = 0.001; Fig. 3A). Participants reported no pain during GVS.

#### Perceived egomotion by passive turntable chair rotation

**Fig. 4:**
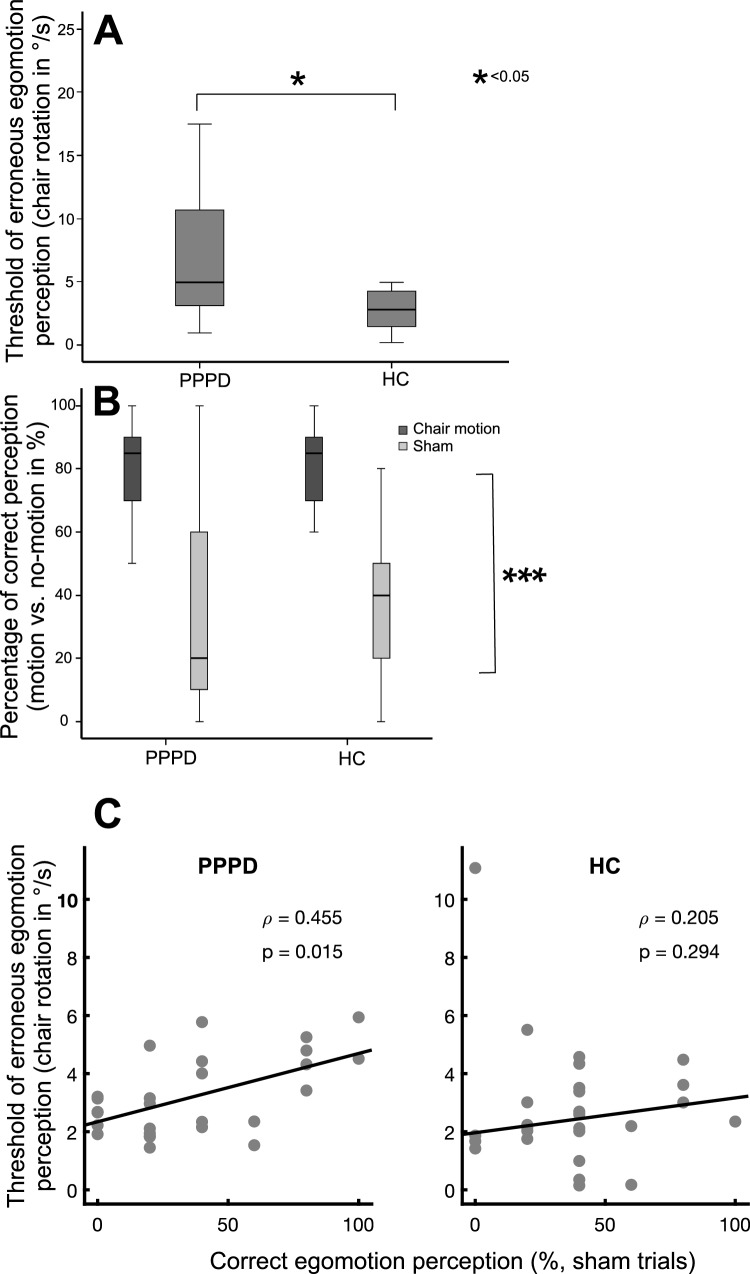
**A** Threshold of erroneous egomotion perception (°/s) display significantly higher values for the PPPD participants. **B** Percentage of correct egomotion perception (motion or no-motion) display no group differences. The percentage of correct motion recognition is much higher during chair rotation compared to the no-motion condition. **C** The threshold of correctly identified egomotion with chair rotation increases with the number (%) of correct responses in the sham condition in PPPD patients but not in healthy control subjects. ***p = 0.001

All participants completed all trials; none of them terminated the experiment due to nausea or dizziness. Threshold is given as chair velocity (°/s) at the time when the patient pressed the button. ANOVA revealed no main effect of GROUP, DIRECTION (vestibular chair rotation) and correct assignment on the motion perception threshold (*p* = 0.287), i.e., there was no difference in threshold of perceived egomotion detection or correct vs. false responses with respect to the direction of stimulation. Accordingly, trials were pooled for further analysis. The level of correct assignments was high in both groups (82.4% in PPPD, 87.5% in HC), i.e., the task was appropriate to study vestibular egomotion perception. In the rotation condition, the threshold of correct passive vestibular motion perception did not differ between PPPD (3.32°/s ± 1.28) and healthy control subjects (3.31°/s ± 2.35) (*Z* = − 1.163; *p* = 0.245) (Fig. 3B). The threshold of about 3°/s indicates that 50% of all subjects decided to notice directional egomotion at about 10 s after the onset of chair rotation (chair acceleration: 0.3°/s^2^, see methods). In the no-rotation condition, the threshold for erroneous perception of the chair motion was larger for PPPD (7.22°/s ± 5.91, *Z* = − 2.636; *p* = 0.008) compared to HC (3.80°/s ± 4.08) [*F*(1,44) = 4.37, *p* = 0.03] (Fig. 4A).

An ANOVA on the number of the participant’s correct assignment showed no significant main effect for GROUP or interaction of GROUP x CONDITION but a main effect of CONDITION [chair rotation vs no rotation; *F*(1, 47) = 114.8, *p* < 0.001; rank transformed data: *F*(1,47) = 100.8; *p* = 0.001], i.e., both groups were able to differentiate chair rotation and no motion (sham condition). However, there were no significant group differences (*p* = 0.929, sham condition: PPPD: 35.7% ± 32.4; HC: 38.6% ± 26.1; rotation: PPPD: 82.2% ± 13.4, HC: 81.8% ± 13.9). Accordingly, the number of erroneous perceptions in the sham condition was not different between groups (PPPD: 64.28 ± 32.4% vs. HC: 61.42 ± 26.1%; Z = − 0.714; *p* = 0.475). (Fig. 4B). The relative number (%) of trials with correct perception (no motion) increased with the threshold of (directional) correct responses for vestibular motion perception in PPPD (Rho = 0.455, *p* = 0.015) but not in HC (Rho = 0.205, *p* = 0.29) (Fig. 4C).

Motion detection threshold by vestibular chair rotation was not correlated with disease duration, age, HADS-A/D, the severity of PPPD (Niigata PPPD score); EQ, ALQ, MSSQ-Short, the NEO_FFI and vestibular motion perception threshold by GVS (*p* always > 0.2).

### Vestibulo-ocular threshold

Due to the very low chair acceleration, vestibulo-perceptual thresholds were indicated by the participants (both groups) before vestibulo-ocular responses were recorded. As we did not continue chair rotation after subjects indicated the egomotion perception (button press), we cannot provide thresholds of vestibulo-ocular thresholds.

## Discussion

The clinical criteria of PPPD encompass active or passive motion and exposure to complex moving visual stimuli (visual dependence) as provocative factors for perceived postural dizziness in PPPD [2]. Over-reliance on visual information for spatial orientation (e.g., visual dependence) has been correlated with the visual vertigo handicap following acute vestibular neuritis [6], i.e., it is characteristic of poorly recovered vestibular neuritis patients. Visual dependence was found to be a predictor for secondary PPPD following vestibular neuritis [5]. This implies changes in the visual motion perceptional sensitivity but thresholds of visual motion perception have not been examined in PPPD yet.

Therefore, we tested the pathophysiological hypotheses that altered perceptional thresholds not only of visual motion but also of passive vestibular egomotion contribute to the development and maintenance of PPPD. As main results, we found in PPPD compared to age-matched healthy control subjects: (1) increased thresholds of visual motion perception, and (2) differential effects of vestibular motion perception with decreased thresholds during GVS but undistinguishable motion thresholds during passive vestibular chair rotation in the dark.

### Visual motion perception

Previous episodes of vestibular stimulation under natural conditions or vestibular disease may disrupt the natural balance control leading not only to heightened self-awareness of postural control by increased limb co-contraction with perceived postural stiffness but also to increased reliance on visual signals [14, 35, 36].

Increased sensitivity to particularly complex visual motion in PPPD might come from low visual motion detection thresholds. In contrast, we found higher visual motion perception thresholds in PPPD patients compared to HC, i.e., patients required more coherently moving random dots to perceive a global visual motion than the age-matched healthy subjects. A poorer complex visual motion recognition, e.g., traffic visual stimuli, may increase anxiety and levels of uncertainty as visuomotor reactions might occur delayed or inappropriate. Concomitant large-field moving surroundings by optokinetic stimulation during functional head impulse tests provoked more reading errors on an optotype display on a computer screen which was not found without optokinetic stimulation [37]. This has been taken as a sign of visual hypersensitivity but it could also imply that complex visual stimulation provokes a stronger distracting stimulus in PPPD compared to healthy subjects. Raised visual contrast thresholds have been found in patients with functional motor disorders, i.e., patients required higher visual contrasts than healthy controls to maintain the same sensory detection sensitivity [38].

The increased threshold of coherent visual motion in our PPPD participants sheds new light on imaging studies investigating visually evoked brain activity and functional connectivity in PPPD showing increased connectivity in networks linking visual and emotion processing areas [39, 40]. The greater reliance on visual rather than vestibular signals for achieving spatial orientation and maintaining balance was suspected to derive from altered visual cortical processing in PPPD [7, 8, 39, 41, 42]. Visual cortex activity increased during simulated vertical visual motion in PPPD in proportion to the dizziness handicap [8] but was found to be normal in a related study with various forms of complex visual stimulation by checkerboard and optokinetic stimulus patterns [35]. It remained unclear whether the structural and functional alteration of the visual cortex activity is the cause or the consequence of an increased sensitivity to complex visual motion in PPPD [5].

Our data, however, could indicate that altered brain activity in response to simulated complex visual stimuli might rather result from abnormal visual motion perception thresholds which was not tested in previous PPPD studies. The influence of visual motion (visual dependence) on vestibular perception has been studied in vestibular neuritis patients using the Rod and Disk test in which patients showed a stronger deviation of the rod tilt during concomitant complex visual stimulation [6] but this has not been reported in PPPD yet. Our RDK stimulation paradigm specifically tested global visual motion which is stimulus-driven and processed bottom up, i.e., the movement of the dots is integrated into a percept of the global movement [29]. This is processed in the dorsal stream of the visual cortex in which visual motion signals from the early visual areas V1 and V2 are integrated in MT/V5 into complex global motion signals. Our paradigm did not test the form of visual features being conveyed in the ventral stream (not involving MT/V5). PPPD subjects usually do not complain about visual blurring (features of visual targets) but are rather disturbed by complex visually moving stimuli such as scenery flowing sideways when viewed from inside a train, and scenery flowing from front to back when riding in a passenger car [35]. The network of MT/V5 and the precuneus which is engaged in visual motion processing appeared to be less connected in patients with postural phobic vertigo [43] which may account for the increased motion detection threshold of our PPPD participants. Contrary to previous assumptions, aggravation of PPPD symptoms by complex visual stimuli might not come from increased reliance on visual processing for postural control but from increased coherent motion detection thresholds. As this was consistently found in our consecutive experimental trials it is probably not related to fluctuations of visual conscious awareness [44].

Interestingly, the threshold of visual motion detection increased with the level of depression in our PPPD participants (HADS-D). Altered visual motion perception of large, high contrast stimuli has been found in depressive patients [45] and suspected to be related to decreased GABA levels in the motion sensitive middle temporal area (MT) of depressed patients [46]. However, recently, global motion processing was found to be intact in depressive patients [47] making it unlikely that depressive features alone account for the increased threshold of visual motion perception in our study. Patients with major depression, however, show abnormal center-surround suppression effects that might indicate a poorer capability of PPPD subjects with depressive symptoms to avoid distraction by concomitant large-field visual stimulation [37].

As other demographic or disease severity factors were not correlated with complex visual motion detection in our PPPD participants, the finding should be replicated. Noticeably, this should also be tested with different sensory modalities as altered sensitivities seem to occur in various sensory domains beyond vision and balance in patients with PPPD [48].

### Vestibular motion perception

Altered vestibular perception is proposed to be a predictive factor for the development of PPPD following vestibular dysfunction [5, 49]. Vestibular perception has been tested in PPPD by GVS [10] and passive chair rotation around the earth-vertical axis [11]. In accordance with our previous study [10], we could confirm lower motion perception thresholds during binaural GVS in this new PPPD cohort indicating a quite robust finding.

Vestibular perceptional thresholds during passive angular horizontal chair rotation in the dark around an earth-vertical axis were reported lower in PPPD compared to healthy control subjects [11]. We could not replicate this finding as there was no group difference of vestibular motion perception in our study with a cohort of similar size. Likewise, Kobel and coworkers also found very recently no worsening of vestibular perceptional thresholds during rotational semicircular canal stimulation but they demonstrated elevated thresholds for roll tilt and linear superior–inferior translation [50]. Generalization, however, is limited as 60% of the small cohort (*n* = 13) of PPPD patients also suffered from vestibular migraine.

There are major differences of our study to the experiments of Wurthmann et al. [11].

First, the duration of stimulation was considerably longer in the study by Wurthmann et al.: they used a lower acceleration (0.1°/s^2^) and it lasted almost 2 min (109 s) in PPPD and even 3 min in healthy participants of slowly increasing acceleration of chair rotation before PPPD participants indicated passive egomotion (thresholds 10.9°/s vs. 29.5°/s). In our study, the median threshold of about 3°/s indicates that 50% of all subjects decided to notice directional egomotion already at 10 s after the onset of chair rotation. Not surprisingly, hardly any participant noticed nausea in our study, in contrast to the subjects in the unusually long stimulation in the study by Wurthmann and coworkers.

Second, in contrast to our study, all participants in the study by Wurthmann et al. were investigated only once in only one (right) horizontal direction to avoid influences due to repetitive measurements. This makes the threshold prone to a prediction error. Repetitive measurements with variable stimulus onset would have reduced this bias as does even more the introduction of a sham stimulus. The direction of perceived motion was not indicated by the button press but subjects were asked afterward for the direction. In our study, participants were asked to indicate their decision on perceived chair rotation not before they were certain about both egomotion perception and its direction. Accordingly, the number of correct responses in our study (86.2%) was higher than in the Wurthmann study (61.5%). Moreover, Wurthmann et al. asked participants during passive chair rotation to rate perceived motion sickness, i.e., there was an acoustic reference for spatial orientation, as there was the request of the investigator to indicate the perceived motion. In contrast, we eliminated all acoustic and visual stimuli which could aid subjects to indicate onset or direction of chair motion.

Third, in the light of the increased attention PPPD patients pay on their postural control, a sham stimulus allows to account for predictive mechanisms [10] but it also sheds light on the pathomechanism of perceived egomotion in rest in PPPD [14]. The frequency of correct responses was significantly higher in motion compared to the sham conditions in both groups, i.e., the error rate (false motion direction assignments) in the sham stimulus condition. This reflects that the low rotatory acceleration in our study is adequate to elicit a motion percept, even before the onset of the vestibulo-ocular responses. In the sham condition, participants are prepared to indicate perceived motion and focus attention on even slight chair rotations. Despite the previous information about unpredictable no-motion (sham) trials participants made significantly less correct responses, i.e., they often perceived the sham condition as egomotion, in both groups. Latency was not crucial as the threshold (median) of correctly recognized egomotion (chair rotation) was not different. Interestingly, this threshold predicted the probability of making false assignments in the sham condition in PPPD, i.e., PPPD patients who are readily able to correctly recognize the direction of chair rotation are prone to perceive egomotion in the sham condition when the chair is not moving at all. This was, however, not reflected by a higher rate of errors (erroneous motion detection) in the sham condition of patients. In turn, PPPD who correctly recognize no egomotion in the sham condition are likely to perceive real chair rotation (egomotion) late, as reflected by the high threshold. This relation was not found to be significant in HC subjects and may, therefore, reflect a feature of PPPD. This might explain why in particular PPPD patients with low real angular rotation detection thresholds for correct responses complain about dizziness at rest as they are at risk of perceiving rest as egomotion. In contrast, PPPD participants with high real angular rotation detection threshold will probably not complain about dizziness in rest but may perceive motion too late in balance-challenging conditions and might not adequately stabilize their posture leading to unsteadiness. This should be tested in future studies.

### Comparison of thresholds of galvanic and rotatory chair vestibular stimulation

GVS is primarily a non-physiological experimental tool to examine the vestibular system. However, it stimulates the vestibular nerve and elicits changes of the resting firing activity in a manner that reliably elicits vestibular egomotion perception and even clinically meaningful postural sway. This perception is clearly dependent on the stimulus intensity allowing to establish sensory-perceptual response curves. Although being an “ecologically non-physiological” stimulus, it helps to detect changes of the thresholds of vestibular perception, most likely playing an important role in PPPD. This role becomes important as thresholds of egomotion perception in PPPD were lower compared to HC during GVS but not during rotatory chair vestibular stimulation, i.e., one would have missed the abnormal perception with rotatory chair investigations only.

Accordingly, both thresholds did not correlate with each other. This reflects different physical properties in terms of the direction of evoked egomotion and the stimulation properties. Our GVS elicits horizontal to-and-fro sway perception, while the angular chair rotation stimulated the rotational VOR with rotatory spinning perception. It stimulates both semicircular canal and otoliths afferents, i.e., gravity perception may be preferentially altered in PPPD [50]. Moreover, stimulation frequencies were different for both techniques and vestibular perception thresholds change with stimulus frequencies [51, 52].

Both stimulations did not elicit vestibular nystagmus with the applied stimulus intensities and duration, i.e., subjects already indicated self-motion perception before the vestibular nystagmus was evoked. We used lower horizontal angular chair accelerations (0.3°/s^2^) compared to previous studies on vestibulo-ocular thresholds (0.51°/s^2^) [33, 53]. While the first study revealed higher egomotion perceptual detection thresholds compared to vestibulo-ocular thresholds in healthy subjects [53] the recent paper showed no differences [33]. Due to the low acceleration in our study, participants perceived the correct egomotion before we could detect vestibular nystagmus. This is in line with the lower vestibular perception threshold by GVS (1 mA) compared to the oculomotor (VOR) threshold (1.6 mA) [54].

In conclusion, we provide some evidence for a distinct sensory-perceptual scaling abnormality making PPPD patients susceptible of perceiving egomotion without any vestibular stimulation.

## Data Availability

The data are available from the corresponding author, upon reasonable request.
